# Commencements

**Published:** 1899-07

**Authors:** 


### COMMENCEMENTS.

### New York Dental School.

The seventh annual commencement exercises of the New York Dental School were held in the Academy of Medicine, New York City, on Friday, May 12, 1899.

An address was delivered by Rev. Frank P. Stoddard.

The degree of D.D.S. was conferred upon the following graduates:

| | | |
|---|---|---|
| Otto Ernst | Edgar F Lewis | Philip Suriani |
| Frank W French | Jacob J Mintz | Geo D Samson |
| Mary Fishman | Anna Rosenson | Maria L Tantum |
| Herman Gruher | Jas R Sutherland | Matilda Sinai |

Total, 12.

### The Columbia University, Washington, D. C., Dental Department.

The annual commencement of this institution was held in April, 1899, when the degree of D.D.S. was conferred upon the following candidates, viz:

| | | |
|---|---|---|
| Arthur Reginald Bennett | Wm. Marshall Kemball | William Edwin Pairo |
| Enrique Cuevas | John R. McChesney | Thomas M. Rice |
| Wm. Cummings Fisher | Stephen D. Pool | William A. Rawson |
| Thomas J. Gates | | Harry Summers Terry |
| Walter Brice Hoofnagle | Total, 13 | William G. Woodford |

### Medico-Chirurgical College of Philadelphia, Department of Dentistry.

The annual commencement of the Medico-Chirurgical College of Philadelphia, was held May 20, 1899, in the Academy of Music. Dr. Wm. Rodman, A.M., M.D., conferred the degree of D.D.S. upon the following candidates, viz:

| NAME. | RESIDENCE. | NAME. | RESIDENCE. |
|---|---|---|---|
| Charles Robert Bain | Canada | George Gould Lawyer | New York |
| Charles Jacob Bingle | Germany | Gabriel Middleton | Pennsylvania |
| Delos VanCola Burges | Pennsylvania | Michael Coleman Ryan, M.D. | Penn. |
| Jas. Middleton Cornyn | Pennsylvania | George Oliver Reed, Ph.G. | Pennsylv'a |
| Emerson Albert Dunbar | Louisiana | Henry Louis Samelson | Tennessee |
| Charles Milton Frantz, V.M.D. | Penn. | John Jacob Stettzer | Pennsylvania |
| Condy C. Gallagher | Pennsylvania | Telesphore Gustave Turcot | Canada |
| August Philip Graf | Indiana | A. F. Wehr | Pennsylvania |
| Harry C. Hadley, Ph.G. | Pennsylvania | Samuel Reynolds Wharton | Indiana |
| John Lincoln Hughes | Pennsylvania | Charles L. Zimmerman | Pennsylvania |
| Edgar Coleman Hoskin | Canada | | |

Total, 21.

### Colorado College of Dental Surgery.

The second annual commencement exercises of the Colorado College of Dental Surgery were held at the St. James Hotel, Denver, Col., on Thursday evening, April 28, 1899.

Dr. W. T. Chambers, dean, addressed the graduating class, and an address was delivered by Dr. A. Stewart Lobingier.

The number of matriculates for the session was twenty-seven.

The degree of D.D.S. was conferred upon the following graduates by Dr. H. A. Fynn, president of the college: Karl S. Hart, of Michigan; Howard T. Chinn, Emma J. Young and Harry T. Ingalls, of Colorado. Total, 4.

### Dental College of the Province of Quebec.

The annual convocation of the Dental College of the Province of Quebec, in affiliation with Bishops University, was held in the Synod Hall, Montreal, on April 12, 1899.

There were seven matriculates for the session.

Chancellor R. W. Heneker conferred the degree of D.D.S. on the following graduates: J. A. Butler, J. K. Cleary, F. W. Henry, W. G. McCabe, and F. L. Wilkinson, all of the Province of Quebec. Total, 5.

### Ohio College of Dental Surgery.

The fifty-third annual commencement exercises of the Ohio College of Dental Surgery were held at the Odeon, Cincinnati, O., on Wednesday evening, April 12, 1899.

The annual address was delivered by Dr. C. L. Work, and the class address by Alvin A. Ranshaw, D.D.S.

The degree of D.D.S. was conferred on the following graduates by Dr. D. W. Clancey, vice-president of the board of trustees:

| NAME | RESIDENCE |
|---|---|
| William S Allen | Indiana |
| Alpha H Barber | Ohio |
| G Foster Bell | Pennsylvania |
| Ulysses O Bikler | Kentucky |
| Frank H Bottenus | Ohio |
| James M Bronaugh | Kentucky |
| John A Chappell | California |
| Carlton W Cragg | Ohio |
| Vernon B Crumbaugh | Ohio |
| Chas L Chamberlin | Illinois |
| Miss Ada A Farr | Michigan |
| Geo T Fette | Ohio |
| James D Ford | Ohio |
| Walter E Fox | Ohio |
| Wm C Geiger | Ohio |
| Horace M Gear | Indiana |
| James M Gilbert | Texas |
| Wm G Hamm | Ohio |
| Howard E Hathaway | Ohio |
| N Graham Higby | Tennessee |
| Harry C Hodges | Kentucky |
| Milton C Hopper | Ohio |
| James W Huffman | Kentucky |
| Chas E Kelsey | Ohio |
| Wm R Kemper | Ohio |
| Wm E Kennedy | Ohio |
| Fred J Kern | Iowa |
| Alvin E Kerst | Arkansas |
| Fred F Kolm | Illinois |
| John R Kuhn | Missouri |
| James C Lehman | Indiana |
| Chas E Martin | Ohio |
| Edwin B McCoy | Indiana |
| John W McGlashan | Ohio |
| James E Meed | West Virginia |
| Frank L Merkle | Ohio |
| Claude Michael | Ohio |
| Homer L Myers | Kentucky |
| Fred M Nixon | Indiana |
| H A Phelps | Ohio |
| Burk Pickett | California |
| Alvin A Ranshaw | Kentucky |
| Wm A Raymond | Ohio |
| Joseph A Reardon | Kentucky |
| T Lawrence Rock | Louisiana |
| Herbert T Schilder | Ohio |
| Fred J Schwierking | Ohio |
| Francis J Shea | Canada |
| Chester W Sheridan | Ohio |
| Edwin B Shrieves | Ohio |
| Alva F Simmons | Kentucky |
| Miss E A Snyder | Ohio |
| Chas G Steen | Kentucky |
| Wm Taft, Jr | Ohio |
| John A Tauber | West Virginia |
| Roger W Taylor | Tennessee |
| Miss Hedy Tcherniac (M.D.S. Geneva) | Germany |
| Kent C Willis | Ohio |
| Samuel C Wolfe | Indiana |
| Fred L White | Ohio |
| Elmer E Young | Indiana |

Total, 62.

### University of Michigan—Dental Department.

The twenty-fourth annual commencement of the Dental Department of the University of Michigan was held June 22, 1899, in University Hall. Commencement oration was delivered by Nicholas M. Butler, LL.D., Columbia College, N. Y., after which the degree of D.D.S. was conferred upon the following candidates by the President of the University:

Hilen D Aldrich

William J Allen

Arthur A Baker

Victor E Bedford

Helmuth P Binzel

Francis L Busch

Will C Butler

Joseph McG Cartwright

Frank P Cattermole

Carroll F Chase

Gilbert A Cotton

Loran S Fleming

George M Freeman

John E Gilbert

Henry W Harvey

Claude E Hathaway

Perry F Hines

Stanley A Horning

Richard J Huyck

Carl A L Johnson

Herbert C King

Wilfred D Kirk

Jay W Kline

William G Law

Jesse Levy

Charles L McKinnis

David C Martin

Sidney Martin

George H Mengel

Clair G Meseroll

Charles J Miller

John Miller

Edgar E Nelson

Fred C Orvis

Harry C Orvis

Charles M Owen

Sidney D Peters

William J Polglase

Francis E Renkenberger

Ray D Robinson

Erwin A Salisbury

Earl W Sanford

Edmund H Shannon

Harry W Sheldon

John F Sortore

Flora Mae Spore

Rene M Squier

Clifford F Stipp

Harrison A Stites, A.M., Hillsdale College

Philip R Thomas

Archibald C Thompson

Loren S Treat

Edward N Trenholme

Fred M Washburn

Benjamin W Wells

Chauncey C Wescott

Frank DeWitt Wilson

Charles A Wise

Alvin O Wright

D.D.Sc. UPON

James R. Davis

Oliver W White

Total, 62.

### Missouri Dental College.

The thirty-third annual commencement of the Missouri Dental College, Dental Department of Washington University, was held at the Fourteenth Street Theater on Thursday evening, April 27, 1899.

Number of matriculates for the session, 113.

The degree of D.M.D. (Doctor of Dental Medicine) was conferred upon each of the following candidates:

| NAME | RESIDENCE | NAME | RESIDENCE |
|---|---|---|---|
| Elwood Alley | Missouri | Sylvester C Nifong | Missouri |
| Henry C Alloway | Missouri | Clarence L Sappington | Missouri |
| Willis B Arthur, M.D | Missouri | Edward Schlagenhauf | Illinois |
| Roy Bay | Missouri | Alexander Scherzinger | Missouri |
| Elliott R Black | Illinois | Ira D Scott | Missouri |
| George W Corder | Missouri | Herman T Spann | |
| Claudius G. Farrow | Arkansas | Henry G Steinmesch | Missouri |
| Stonewall J Ferguson | Missouri | Charles E Stephens | Alabama |
| Henry R Hoffman | Missouri | Edwin W B Temm | Missouri |
| Merle R Hopkins | Illinois | Edward W Walker | Missouri |
| Porter Kendall | Missouri | Wilson R Weber | Texas |
| Herman M Lansberg | Missouri | John H Wild | Missouri |
| Philip J Lenhard, Jr. | Missouri | Rvdolph F Wild | Missouri |
| Charles R Mockbee | Missouri | Raymond LaC Willett | Iowa |
| Henry C Mueller | Illinois | Eugene D Wurtz | Illinois |

Total, 30.

### Western Reserve University Dental College.

The commencement exercises of the Western Reserve University Dental College were held June 15th, in Beckwith Memorial Church, Cleveland, O. President Charles Gilmer, of Johns Hopkins University delivered a suitable address. President Charles F. Shiving, D.D., LL.D., conferred degrees upon the following candidates:

Ross Willis Andrews

Arthur Dick Apple

Almon Lee Atwater

Liscomb Dalzell Auxter

Homer Aaron Baldwin

Varney Edward Barnes

George Bridgeman

William Edward Costello

Harry Poe Eaby

Leigh Lawrence Finch.

Samuel Taylor Gilmore

Emanuel Grossman

Frank Joseph Gunn

Jay Carleton Kelley

John Mistr

Chas C Mottinger

Jay Kimmel Nash

Edward Lee Norton

Frank Lyman Olds

James Alfred Rupert

Frank Watson Stevenson

Cameron R Stewart

Charles Edward Taylor

Oscar John Van Dorston

Samuel M Weaver

John Barton Webber

Charles Nelson White

Douglas Austin Wright

Dan Hendrix Ziegler

Total, 29.

### College of Physicians and Surgeons of San Francisco.

The annual commencement of the dental department of the College of Physicians and Surgeons of San Francisco was held July 12, 1899, in the California Theater. The degree of D.D.S. was conferred by Prof. J. R. Laine, M.D., upon the following candidates:

William H Armstrong

Albert Minor Barker

Allen McLean Barnes

Claude Ransome Basford

Elmer H Benjamin

W E Broadwater

William Edgar Brooks

Robert Clinton Brower

Edward Evens Brown

Oreian B Burns

Alfred Cane

John Chalfant

James Fenton Chappell

William H Clarke

George Lingow Cool

Albert Nassau Cooper

Louis Theodore Cranz

John William Creagh

George Eaton Daniels

Carl W Dewlaney

Xavier Dodel, M.D.

Hudson de Wolf Dodge

C S Duckett

Benjamin F Edwards

Edward George Eisen

W A Ellis

George C Farmer

Lawrence M Finigan

Thomas Fletcher

William S Fowler

H A Frederick

Chester Clyde Gilbert

Carey D Gorton

Adolph M H Griesser

Jesse W Hamilton

F J Harvey

Otto Albert Hasslinger

Frank Thomas Heacock

William Rema Heacock

John Cornelius Hennessy

Thomas Locker Hill

Albert Oliver Hooker

Evan L Jones

Max Victor Kempe

Calvin William Knowles

Warren Lafayette Lackey

Delmar H Latimer

Walter Frederick Lewis

John O Laughlin

Antonio Martin Luz

A E Macdonald

Arthur Bell Mayhew

Albert William McKenzie

William F McLaughlin

Rodolph Wallace Meek

Alvin Fox Merriman, Jr.

William Cole Merriman

Albert Meyer

Charles Wesley Mills

Harry Everett Minor

George Harvey Ohea

James Nicholas Powell

Robert Henry Power

John Robertson

Mack Jay Seeley

Henry Sichel

A L Simpson

Charles Leipt Smith

Charles Fred Sloat

Edward Ernest Sparhawk

Thomas E Strong

Thomas Xavier Sullivan

Sand J Symmons

Antoine W Taylor

Frederic Teague

Luther A Teague

Clarence Bruce Tennyson

George W. Thurston

William Osbourne Toye

James William Walsh

Parker S Wilbur

Oliver Thomas Wilson

Total 82.

### Indiana Dental College.

The annual commencement exercises of the Indiana Dental College were held in English's Opera House, Indianapolis, Ind., on Thursday, April 6, 1899.

The annual address was delivered by John N. Hurty, M.D., Ph. D.

The degree of D.D.S. was conferred upon the following graduates by President Burris A. Jenkins, of the University of Indianapolis:

| NAME. | RESIDENCE. | NAME. | RESIDENCE. |
|---|---|---|---|
| William H Allen | Kansas | J W Hedderich | Indiana |
| John D Altenburg | Indiana | Geo A Hubbard | Indiana |
| Frank P Anderson | Indiana | Christian F Kuhn | Indiana |
| Daniel A Andrews | Indiana | Albert H Littlefield | Indiana |
| Frank W Bell | Indiana | Wilfred J Lawrie | California |
| Roy F Bennett | Ohio | Maurice J Lucas | Indiana |
| Benjamin S Binford | Indiana | Nicholas E Lutz | Indiana |
| Alfred Braddock | Indiana | Harry E Lyman | Kansas |
| Ernest R Burket | Indiana | Samuel A Martin | Indiana |
| W G Campbell | Washington | Jacob F. Moyer | Ohio |
| Perley H Chadwick | Michigan | Thomas G Noughton | Indiana |
| John H Cheek | Indiana | Chas T Noble | Pennsylvania |
| Karl Christofferson | Wisconsin | Samuel Ordetx | Cuba |
| J Frank Cofield | Indiana | Wm V Owen | Kentucky |
| Clinton E Conley | Iowa | Arthur B Phillips | Indiana |
| E M Conway | Indiana | Will H Porter | Illinois |
| James W Corson | Indiana | Cortland R Price | Ohio |
| Charles W. Day | Indiana | Elbert C Repass | Ohio |
| Harrison E Dempsey | Washington | Jesse W Stage | Indiana |
| Charle E Eads | Indiana | Griffin S Stephenson | Indiana |
| W [illegible] Eckelman | Indiana | Archibald Stover | Pennsylvania |
| Godfrey D Eggeman | Ohio | Lee R Talbert | Minnesota |
| Wm A Fishburn | Washington | Earl C Thompson | Indiana |
| Harry A Fisher | Indiana | Robert S Thompson | Indiana |
| Benton O Flora | Indiana | Wm H Thompson | Illinois |
| Forrest E Freeman | Indiana | Fred W Turley | Indiana |
| Wm M Garver | Indiana | Alva H Unthank | Indiana |
| Frank L Gilman | Kansas | E F Van Osdol | Indiana |
| William H Green | Indiana | L E Van Osdol | Indiana |
| Harry W Gregg | Indiana | Frank A Wildason | Indiana |
| Geo R Guthrie | Indiana | L E Wineinger | Indiana |
| Walter D Haisley | Indiana | | |

Total, 63.

### Ohio Medical University—Department of Dentistry.

The seventh annual commencement of the Dental Department of the Ohio Medical University was held April 18, 1899, at the Great Southern Theater, Columbus, O.

The number of matriculates for the session was one hundred and fifty-three.

The degree of D.D.S. was conferred upon the following:

| | | |
|---|---|---|
| H S Barrick | E L Murphy | W E Jackson |
| J H Bristor | D L Rankin | R F Leslie |
| J W Dixon | G W Reighner | H M McDonald |
| J A Douglass | Philip Rowland | Harry McLaughlin |
| F P Haverfield | W W Scales | V I Miller |
| E W Iden | L H Black | W Chas Neff |
| F G Jackson | R B Cochrane | E C Reed |
| J T Lewis | C E W Downie | J A Rockey |
| G H McFarland | G I Gunckie | J C Stover |
| A O McLaughlin | W B Horton | H D Thomas |

Total, 30.

### Miami Dental College.

The annual commencement exercises of the Miami Dental College was held in the Grand Hotel Cincinnati, Ohio, on Friday evening, April 21, 1899.

The number of matriculates for the session was nine.

The degree of D.D.S. was conferred on the following graduates by Mr. Wm. Schuhardt, president of the board of trustees: Fred C. Addison, Wm. A. Cooper, Edwin E. Harcourt, and Frank C. King, all of Ohio. Total, 4.

### Keokuk Dental College.

The annual commencement exercises of the Keokuk Dental tal College (Dental Department of Keokuk Medical College) were held in the Opera House, Keokuk, Ia., on Monday, March 20, 1899.

The number of matriculates for the session was thirty-five.

The degree of D.D.S. was conferred upon the following graduates by Geo. F. Jenkins, A.M., M.D., president of the college: Lloyd S. Lourie and John M. Hobbs, of Iowa, and James H. Walker, of Kansas. Total, 3.

